# A Study of the Relationship Between Objective Tests to Diagnose Erectile Dysfunction and Markers of Cardiovascular Disease

**DOI:** 10.3390/jcm13216321

**Published:** 2024-10-23

**Authors:** Maurizio De Rocco Ponce, Claudia Fabiana Quintian Schwieters, Juliette Meziere, Josvany Rene Sanchez Curbelo, Guillem Abad Carratalá, Eden Troka, Lluis Bassas Arnau, Eduard Ruiz Castañé, Maria José Martinez Barcina, Osvaldo Rajmil

**Affiliations:** 1 Fundació Puigvert, Instituto de Investigaciones Biomédicas Sant Pau (IIB-Sant Pau), Universitat Autònoma de Barcelona, 08041 Barcelona, Spain; cquintian@fundacio-puigvert.es (C.F.Q.S.); jrsanchez@fundacio-puigvert.es (J.R.S.C.); lbassas@fundacio-puigvert.es (L.B.A.); eruiz@fundacio-puigvert.es (E.R.C.); mjmartinez@fundacio-puigvert.es (M.J.M.B.); orajmil@fundacio-puigvert.es (O.R.); 2 San Luigi Gonzaga University Hospital, 10043 Orbassano, Italy; juliette791@gmail.com; 3 Hospital Provincial Castellón de la Plana, 12002 Castellón de la Plana, Spain; gabadcar@gmail.com; 4 Facoltà di Medicina e Chirurgia, University of Padua, 35127 Padova, Italy; trokaeden@hotmail.com

**Keywords:** erectile dysfunction, Penile Color Doppler Ultrasound, Nocturnal Penile Tumescence and Rigidity test

## Abstract

**Background:** Erectile dysfunction (ED) can stem from various organic and functional causes but is often linked to vascular health and cardiovascular disease. Limited data exist on how cardiovascular disease markers correlate with objective ED tests like the Nocturnal Penile Tumescence and Rigidity (NPTR) test and Penile Color Doppler Ultrasound (PCDU). **Methods**: A prospective observational study was performed, and 58 men with ED were assessed using the International Index of Erectile Function-15 (IIEF-15), NPTR test, and PCDU. Peripheral vascular health was evaluated through carotid intima-media thickness (cIMT) and brachial flow-mediated dilation (FMD). **Results:** Out of the participants, 44 had normal NPTR results, while 14 had abnormal results. The group with abnormal NPTR results was significantly older and had higher rates of hypertension and diabetes. Although the IIEF-15 scores were similar between the two groups, those with abnormal NPTR results had a lower peak systolic velocity (PSV) and a higher prevalence of impaired PSV. Correlations between the IIEF, NPTR, PCDU, and peripheral vascular markers lost significance after the age adjustment. **Conclusions:** This study suggests that abnormal NPTR results, combined with cardiovascular risk factors, may signal vascular ED and generalized vasculopathy, highlighting the need for cardiovascular assessment. An accurate ED diagnosis should integrate clinical evaluation with multiple tests while considering aging as a key risk factor.

## 1. Introduction

Erectile dysfunction (ED) is defined as the persistent inability to obtain or maintain a penile erection sufficient for satisfactory intercourse [1]. It is the most frequent male sexual dysfunction alongside premature ejaculation. ED affects men of all ages, reaching a prevalence of over 70% by the age of 80 [2] with a significant impact on sexual quality of life and quality of life. Furthermore, it serves as a cardiovascular risk marker with important implications for general health [3,4].

ED is not a single entity but rather a symptom related to multiple potential causes. While some etiologies of ED, such as penile trauma or those secondary to prostate surgery, are clear, diagnosis in most patients can be challenging, requiring a comprehensive clinical workup to identify the underlying cause.

Schematically, we could distinguish between two main categories: organic ED, which mainly arises from vascular, hormonal, or neurological impairment, and on the other hand, psychogenic causes of ED (or “functional ED”). However, it is known that commonly, a mixed spectrum of etiologies can be found, varying in proportion with age [5]. Actually, the patient’s age represents one of the most important factors to predict a specific type of ED because psychogenic ED is much more common among young men, while in older men, vascular ED is the most prevalent. Generally speaking, we know that vascular ED is the most common form of ED, and it can account for up to 70% of all cases [6]. Furthermore, ED serves as an early manifestation of generalized vascular impairment, taking a role of sentinel symptom of subclinical cardiovascular disease (CVD) and as an independent risk factor for major cardiovascular events (MACEs). Actually, the altered function of the corpus cavernosum is tightly associated with the cardiovascular system’s function. ED and CVD share similar risk factors, so their interplay is tight and bidirectional [7,8].

Penile Color Doppler Ultrasound (PCDU) and the Nocturnal Penile Tumescence and Rigidity (NPTR) test are objective tests for erectile dysfunction diagnosis that provide different information. PCDU assesses penile hemodynamics, while the NPTR test measures the quality of nocturnal involuntary erections during the Rapid Eye Movement (REM) phase of sleep [9,10]. Currently, PCDU is considered one of the best tools to assess penile vascular impairment [10]. The NPTR test, on the other hand, assesses the intact mechanism or inappropriate nocturnal erections without the administration of vasoactive drugs or the intervention of the observer. It is considered a useful tool to differentiate between organic and psychogenic ED [11]. Despite the acknowledged relationship between ED and CVD, in the literature, specific data about the relationship between the NPTR test, PCDU, and markers of cardiovascular disease are scarce. When the NPTR test or PCDU is performed, the information provided is of help for ED diagnosis and definition, but their value as clues for the presence of an underlying CVD is not established. In particular, we do not know their association with other well-established CVD markers.

The aim of this study is to investigate the relationship between the NPTR and PCDU assessments in men with ED and their relationship with markers of cardiovascular disease.

## 2. Materials and Methods

We conducted a prospective observational study involving patients from the Andrology Department. Patients’ inclusion criteria were men aged 18 years or more consulting for erectile dysfunction lasting 3 months or more. Men with iatrogenic ED (e.g., ED after prostate surgery), Peyronie disease, severe neurological or psychiatric conditions, severe kidney or liver failure, or oncological conditions were excluded. None of the participants were treated with any erection-inducing medication prior to the finalization of the study protocol.

Detailed medical history with a complete physical examination and blood tests were collected for all patients.

All participants completed the International Index of Erectile Function-15 (IIEF-15) questionnaire [12] and the International Prostatic Symptoms Score (IPSS) questionnaire [13]. The severity of ED was classified into five categories according to the IIEF-EF score as follows: no ED (EF score 26 to 30), mild (EF score 22 to 25), mild to moderate (EF score 17 to 21), moderate (EF score 11 to 16), and severe (EF score 6 to 10) [14].

Every patient underwent an NPTR test, PCDU, and a peripheral vascular morpho-functional assessment. A 3-night NPTR test was performed with the RigiScan^®^ Monitor (Dacomed Corporation, Minneapolis, MN, USA). A record of at least one episode of rigidity with 60% rigidity lasting for 10 min or more obtained in 3 consecutive registered test nights was defined as normal [15].

For PCDU, a high-resolution color Doppler ultrasound (Siemens ACUSON NX3 Elite, Munich, Germany) equipped with a 4.0–12.0 MHz probe (axial resolution < 0.1 mm) was used. PCDU was performed after an intracavernous injection of alprostadil 10 mcg (Caverject^®^). During the following 20 min, the PSV was measured at the peno-scrotal junction until stabilized, and a PSV ≥ 35 cm/s was considered normal [10,16,17,18]. No patient required a re-dose to perform a proper PCDU procedure, and no one needed a reducing treatment.

Finally, to assess the peripheral vascular status, we used two different markers: an ultrasound measurement of the intima-media thickness at the carotid arteries (cIMT) and a post-ischemic dilation at the brachial artery (flow-mediated dilation, FMD) using the same ultrasound device used for the PCDU. For the cIMT, we measured the distance between the lumen and the adventitia in three different points of both carotids, and the mean value was used for the statistical analysis. We considered a cIMT of ≥1 mm as impaired [19]. The FMD measure was performed in a fasting condition in the morning, in a supine position, with a blood pressure cuff on the patient’s right arm and after a relaxing period of 15 min in a temperature-controlled room. In the brachial artery, in the antecubital fossa, a measurement of the diastolic diameter of the artery was obtained. The cuff inflated up to a super-systolic pressure of around 200 mmHg for 5 min; after, the cuff was deflated, and the diastolic diameter of the artery was measured during the post 40–60 s. We express FMD as the maximum relative increase (%) in the artery diameter over baseline. As suggested by the literature, we considered an FMD value ≥ 6.5% as optimal for an endothelial function, while an FMD value ≤ 3.1% defined an impaired endothelial function [20].

Statistical analysis was performed using SPSS statistics software for Windows (Version 23, SPSS Inc., Chicago, IL, USA). The Kolmogorov–Smirnov test was used to test the normal distribution; as normal distribution was not confirmed for all variables, we used nonparametric tests. Continuous variables are expressed as median and 25th–75th percentile interquartile interval.

We divided our patients into two groups based on the NPTR results as part of data analysis. Comparison between subgroup was performed with the Wilcoxon–Mann–Whitney test. Categorical variables are expressed as frequencies and percentages and were compared between groups using Pearson’s chi-squared test.

To investigate the relationship between data obtained from the peripheral vascular assessment, the NPTR test, and the PDCU, we conducted a correlation analysis among morpho-functional data obtained in the penile and peripheral vascular assessments (PSV, cIMT, and FMD) and data from the NPTR record summary (number of events [i.e., erections], maximum event duration, and base and tip rigidity). The relationship between continuous variables were evaluated by Spearman’s correlation coefficient (ρ). All reported probability values are two-tailed, and a value of *p* < 0.05 was considered statistically significant. A statistical power calculation was performed using MedCalc statistical software (Version 23.0). As previous studies on patients with erectile dysfunction showed a mean cIMT of 0.70 ± 0.18 mm [21], a difference of 30% was assumed to be significant to ensure an adequate statistical power of at least (1 − β) = 80% and α = 5%, and the minimum sample size obtained was 24 patients.

## 3. Results

Fifty-eight consecutive patients were included, with a median age of 47.0 (35.7–57.2) years and an ED duration of 3.0 (1.2–9.6) years. The median IIEF-EF score corresponded to severe ED (9.0 [5.5–16.0] points), while the median IPSS score was normal (7.0 [1.7–14.0] point). Of the 58 patients, 44.8% were active smokers, 17.2% had hypertension, 15.5% had diabetes mellitus, 20.7% had dyslipidemia, and 6.9% had a history of previous cardiovascular disease (CVD). Their median body mass index was 25.0 (23.1–26.7) kg/m^2^. The general characteristics of the study participants are summarized in Table 1.

The studied group of patients presented a PSV after alprostadil intracavernous administration of 61.0 (47.2–79.0) cm/s that is considered a normal cavernous blood flow, a cIMT of 0.7 (0.6–0.9) mm that is considered normal, and a FMD response over a baseline of 6.9 (1.9–12.5)%, which indicates normal endothelial function.

Forty-four patients had a normal NPTR result, and fourteen had an abnormal NPTR record. We compared the two groups regarding general clinical characteristics, blood tests, and vascular assessment results. The group with altered NPTR results were significantly older than the patients with normal NPTR results (55.0 vs. 44.1 years; *p* = 0.002), with a higher prevalence of hypertension (50.0 vs. 6.8%, *p* = 0.001) and with a longer hypertension duration (7 years vs. 1 year; *p* = 0.035). Diabetes mellitus was significantly more prevalent as well (42.9 vs. 6.8%; *p* = 0.004) with higher blood triglyceride levels (147 vs. 88 mg/dL; *p* = 0.002). No significant difference was found in the CVD prevalence between the two groups. On the other hand, we also calculated the atherogenic index (AI) for our study population. The mean AI was 0.38 (0.17–0.57), corresponding to an increased risk. As expected, the group with abnormal NPTR results presented a significantly higher AI when compared with the patients with normal NPTR results (0.68 vs. 0.32; *p* = 0.004) [22,23].

The IIEF-ED questionnaire was not statistically different between the two groups (10 vs. 9 points; *p* = 0.649). On the other hand, the PCDU demonstrated a significantly lower PSV in patients with abnormal NPTR results compared with the group with normal NPTR results (49.6 vs. 66.1 cm/s; *p* = 0.002). Fifty patients presented a PSV within the normal range, while eight patients presented a PSV < 35 cm/s. We analyzed the distribution of the patients with a pathological PSV and found a higher prevalence among patients with an altered NPTR result, but this difference did not reach a statistical significance. A second age-dependent cut-off was used to define normal vs. pathological PSV [16]. Using this age-dependent threshold, 12 patients had a pathological PSV, and the prevalence among patients with abnormal NPTR results was statistically higher than among those with normal NPTR results (50.0 vs. 11.4%, *p* = 0.005). Finally, the peripheral vascular assessment showed no significant differences as regards the cIMT and FMD between the two groups (see Table 2).

The relationship between the PCDU and the NPTR results with markers of the peripheral vascular assessment was explored. We found a statistically significant correlation between the PSV and the cIMT (−0.352, ρ = 0.009) and the maximum number of events in the NPTR record (+0.344, ρ = 0.012). The PSV did not correlate with other NPTR record data but presented other significant correlations with age (−0.540, ρ < 0.001), BMI (−0.402, ρ = 0.002), IIEF-EF score (+0.354, ρ = 0.007), glycemia and HbA1c (−0.407, ρ = 0.006 and −0.451, ρ = 0.007), total cholesterol (−0.341, ρ = 0.029), and triglycerides (−0.339, ρ = 0.030). Finally, the FMD had no significant correlation with the PSV but had a significant correlation with the cIMT (−0.360, ρ = 0.011). When corrected for age, all of these associations lost their statistical significance.

## 4. Discussion

A proper diagnostic workup is crucial when dealing with erectile dysfunction because a correct etiology assignation ensures the patient has the best treatment options and therefore the best outcomes. Moreover, the altered function of the corpus cavernosum is tightly associated with the cardiovascular system’s function and shares similar risk factors. For this reason, identifying a vasculogenic ED may help uncover a subclinical cardiovascular disease [7,8].

Usually, ED diagnosis relies on accurate medical history collection and the use of validated questionnaires. Blood tests may be required as well when other conditions associated with ED are suspected (e.g., hypogonadism). Few objective examinations are also available to assess erectile function, with the PCDU and NPTR being two of these.

The use of the NPTR test has been proposed as a simple and inexpensive diagnostic tool in recent decades [9,24]. This test would be of help in the differential diagnosis between organic and psychogenic ED as a positive (normal) NPTR result strongly suggests a psychological etiology of the problem; conversely, a negative (abnormal) NPTR result suggests organic ED. This method is claimed to have some limitations related to result interpretation which must be carefully evaluated within the clinical context; high rates of record artifacts and false negatives must also be considered [25]. For example, good sleep quality is required to perform a good NPTR test, so obstructive sleep apnea syndrome (OSAS) is, at the same time, a comorbidity associated with ED [26] and a confounder for the NPTR test. Moreover, just like ED, OSAS is related with CVD [27]. In our small study population, we had no patients with such diagnosis and no patient was reported with impaired sleep during the three-night nocturnal penile rigidity ant tumescence test.

Furthermore, the interpretation criteria of the NPTR results were proposed [15,28] but not universally accepted, and there is still some debate around rigidity results [29]. Moreover, no NPTR device has received official approval from the FDA for ED diagnosis or testing.

Another objective test used for ED diagnosis is PCDU, which is considered helpful in assessing penile hemodynamics and exploring whether there is a compromised vascular component [10]. The exam provides information about the cavernous artery and the cavernous penile structure and function. Furthermore, it allows for the visualization of alterations such as in Peyronie’s disease and the impairment of the hemodynamic response to a pharmacological stimulus [30,31].

In the present study, in addition to an NPTR test and PCDU, each patient underwent a peripheral vascular assessment using carotid artery ultrasound to measure the carotid intima-media thickness (cIMT) and brachial artery ultrasound to perform a flow-mediated dilation test (FMD). These makers correlate with CVD since a high cIMT is considered a strong predictor of incipient vasculopathy [16,25,28], while the FMD assesses endothelial-dependent vasodilation, providing important information about endothelial health [32]. FMD is also claimed to be sensitive to the effect of therapeutic interventions [33,34] independently from other cardiovascular risk factors [35].

The patients studied exhibited overall normal penile and peripheral vascular assessments; however, the comparison between groups revealed significant findings. In fact, those with abnormal NPTR results showed significantly lower PSV than patients with normal NPTR results. This finding was consistent with their statistically higher prevalence of cardiovascular risk factors, such as an older age and a higher prevalence of hypertension and diabetes mellitus. Interestingly, the mean PSV in both groups was within the normal range (PSV > 35 cm/s) [10], and the prevalence of men with a pathologic PSV (<35 cm/s) was not statistically different between the two groups. Moreover, no significant differences were found in the cIMT and FMD between the two groups. On the other hand, patients with abnormal NPTR results showed higher cIMT and lower FMD values without reaching statistical significance. The same occurred with the prevalence of previous CVD as it was higher in the group with abnormal NPTR results, but without statistical significance, which could be explained by the small sample studied.

The correlation analysis revealed a significant association of the cIMT with FMD and with PSV. In turn, the PSV presented significant correlations with age, BMI, glycemia, HbA1c, total cholesterol, and triglycerides (all known cardiovascular risk factors which are linked to vascular ED). A key point of the correlation analysis is that, when a correction for age was applied, the overmentioned associations lost their statistical significance. One could speculate that all data obtained are in fact co-variables depending on age as a common worsening factor for penile function and for the progression of co-morbidities. This point of view could explain that any independent correlations among these data disappear when corrected for age. The present results suggest the importance of age in the development of ED, as supported by previous studies in the literature with larger sample sizes [36,37].

Bearing in mind the importance of age, we decided to analyze our data from the PCDU according to a previously published PSV cut-off which considers the normal PSV as an age-dependent variable [16]. With this other PSV threshold to define, whether the PSV is normal or not, the difference in the prevalence of patients with a pathological PSV between patients with normal or abnormal NPTR results increased and reached a statistical significance. It is not easy to explain this finding. We know that the PSV threshold of 35 cm/s has been demonstrated in correlation studies on ED and cardiovascular disease (CVD). However, our patients presented a very low prevalence of CVD (6.9%) and probably a mild vascular impairment, so it could be speculated that the age-dependent PSV threshold may be more sensitive to subtle alterations in penile hemodynamics, and therefore, it is more efficient to identify patients with an early endothelial dysfunction as we previously described [38].

Regarding the IIEF-EF scores, they correlated with the PSV obtained in PCDU as expected, showing how the cavernous arteries’ function can reflect on the reported erectile capacity. However, while the IIEF-EF showed a median score of nine points, which belong to the severe ED range, the median PSV of 61 cm/s was in the normal range. Moreover, when comparing patients with normal vs. abnormal NPTR results, the IIEF-EF did not show any statistical difference. These findings highlight the complexity of ED etiology and the limitations of the IIEF-EF score in defining organic ED as many men with psychogenic ED can have low scores as well [14]. We might argue that a self-reported questionnaires such as the IIEF score can give us information about the perceived erectile function, which may not correspond to the objective erectile capacity of the penis [39].

Finally, it is of interest that there has not been any correlations found between subjective or objective tests for erection (i.e., the IIEF-EF score, the PSV in PCDU, or the NPTR assessment) and the IPSS score or the total testosterone level. However, it must be said that our population presented normal IPSS scores and testosterone values.

The main strength of this study is the utilization of objective methods to evaluate erectile function and vascular health as opposed to other studies that primarily rely on questionnaires.

The main limitation of this study is the sample size, which may have affected the capacity to show statistical significance in some results. Moreover, the FMD performed manually is subjected to some variability and measurement errors. Future studies may use automatic methods to better assess endothelial function [40]. Finally, part of the data were analyzed using an age-dependent threshold to classify the PSV of our patients; this criterion has been published in recent years [16] but never validated by other investigation groups. We hope that a larger independent study will be conducted to better investigate this point.

## 5. Conclusions

The present study suggests that abnormal NPTR results combined with other cardiovascular risk factors may indicate the presence of vascular ED and generalized vasculopathy. Abnormal NPTR results in patients with erectile dysfunction should prompt a cardiovascular risk assessment to be performed. An ED diagnosis must be clinical and based on multiple instruments, with aging emerging as a crucial risk factor.

## Figures and Tables

**Table 1:** The characteristics of the study population (N = 58).

Age (years)	47.0 (35.7–57.2)
ED duration (years)	3.0 (1.2–9.6)
IIEF-EF (score)	9.0 (5.5–16.0)
Mild ED (N, %)	3 (5.1)
Moderate ED (N, %)	12 (20.7)
Moderate to severe ED (N, %)	11 (19.0)
Severe ED (N, %)	32 (55.2)
IPSS (score)	7.0 (1.7–14.0)
BMI (kg/m^2^)	25.0 (23.1–26.7)
Smoke (N, %)	26 (44.8)
Hypertension (N, %)	10 (17.2)
Hypertension duration (years)	2.0 (0.5–8.9)
Diabetes mellitus (N, %)	9 (15.5)
Diabetes duration (years)	10.0 (2.1–14.6)
HbA1c (%)	5.4 (0.7)
Dyslipidaemia (N, %)	12 (20.7)
CVD (N, %)	4 (6.9)
eGFR (ml/min)	95.0 (85.0–103.0)
Total cholesterol (mg/dL)	183 (159–206)
HDL cholesterol (mg/dL)	45 (37–52)
LDL cholesterol (mg/dL)	102 (89–132)
Triglycerides (mg/dL)	99 (70–134)
Total testosterone	17.2 (13.8–21.8)
Calculated free testosterone (pmol/L)	331 (263–401)

ED: erectile dysfunction; IIEF-EF: International Index of Erectile Function (Erectile Function domain); IPSS: International Prostatic Symptoms Score; BMI: body mass index; HbA1c: glycated hemoglobin; CVD: diagnosed cardiovascular disease; eGFR: estimated Glomerular Filtration Rate. All data are expressed as median (25th–75th percentile interquartile interval) or as frequency (percentage).

**Table 2:** Normal versus abnormal nocturnal erections.

	Normal NPTR (N = 44)	Abnormal NPTR (N = 14)	*p*-Value
Age (years)	44.1 (34.2–55.0)	55.0 (49.5–64.2)	0.002
ED duration (years)	3.0 (2.0–8.0)	5.0 (2.7–9.0)	0.059
IIEF-EF (score)	10 (6–16)	9 (4–14)	0.649
Mild ED (N, %)	3 (6.9)	0 (0)	0.983
Moderate ED (N, %)	8 (18.2)	4 (28.6)	0.403
Moderate to severe ED (N, %)	9 (20.4)	2 (14.3)	0.608
Severe ED (N, %)	24 (54.5)	8 (57.1)	0.864
IPSS (score)	5 (1–11)	8 (2–15)	0.357
Smoke (N, %)	21 (47.7)	5 (35.7)	0.543
Hypertension (N, %)	3 (6.8)	7 (50.0)	0.001
Hypertension duration (years)	1 (0.5–1.5)	7 (2–12)	0.035
Diabetes mellitus (N, %)	3 (6.8)	6 (42.9)	0.004
Diabetes duration (years)	1 (0.5–1)	10 (4–16)	0.136
Dyslipidemia (N, %)	8 (18.2)	4 (28.6)	0.457
CVD (N, %)	2 (4.5)	2 (14.3)	0.243
Atherogenic index	0.32 (0.12–0.58)	0.68 (0.38–0.72)	0.004
BMI (kg/m^2^)	24.9 (23.7–26.5)	24.5 (22.9–27.3)	0.778
Glycemia (mg/dL)	90 (84–97)	98 (96–123)	0.004
HbA1c (%)	5.4 (5.1–5.7)	6.2 (1.3)	0.210
eGFR (ml/min)	93 (85–103)	99 (66–102)	0.622
Total cholesterol (mg/dL)	182 (154–204)	188 (175–242)	0.157
Triglycerides (mg/dL)	88 (68–123)	147 (133–237)	0.002
HDL cholesterol (mg/dL)	45 (36–53)	44 (39–51)	0.947
LDL cholesterol (mg/dL)	105 (88–136)	97 (92–115)	0.741
Total testosterone (nmol/L)	17.8 (13.7–21.9)	16.8 (13.6–20.8)	0.845
Calculated free testosterone (pmol/L)	356 (269–411)	294 (254–345)	0.194
PSV (cm/s)	66.1 (53.9–90.0)	49.6 (33.9–58.9)	0.002
EDV (cm/s)	5.2 (0.6–12.0)	8.8 (0.6–12.8)	0.614
Resistance Index	0.90 (0.81–1.01)	0.81 (0.71–1.00)	0.075
PSV < 35 cm/s (N, %)	4 (9.1)	4 (28.6)	0.086
PSV < age-dependent cut-off (N, %)	5 (11.4)	7 (50)	0.005
cIMT (mm)	0.7 (0.6–0.9)	0.8 (0.7–0.9)	0.174
FMD (%)	7.2 (2.4–12.9)	6.6 (1.7–10.7)	0.434
FMD < 6.5%	17 (42.5)	5 (41.7)	0.959
FMD < 3.1%	10 (25)	4 (33)	0.690

ED: erectile dysfunction; IIEF-EF: International Index of Erectile Function (Erectile Function domain); NPTR: nocturnal penile tumescence and rigidity; IPSS: International Prostatic Symptoms Score; CVD: diagnosed cardiovascular disease; BMI: body mass index; HbA1c: glycated hemoglobin; eGFR: estimated Glomerular Filtration Rate; PSV: Peak Systolic Velocity; EDV: End-Diastolic Velocity; cIMT: carotid intima-media thickness; FMD: flow-mediated dilation. All data are expressed as median (25th–75th percentile interquartile interval) or as frequency (percentage).

## Data Availability

The data can be provided by the corresponding author upon reasonable request.
